# Host‐pathogen kinetics during influenza infection and coinfection: insights from predictive modeling

**DOI:** 10.1111/imr.12692

**Published:** 2018-08-11

**Authors:** Amber M Smith

**Affiliations:** ^1^ University of Tennessee Health Science Center Memphis TN USA

**Keywords:** Bacterial infection, coinfection, infectious diseases, lung, mathematical model, model validation, monocytes/macrophages, T Cells, virus infection

## Abstract

Influenza virus infections are a leading cause of morbidity and mortality worldwide. This is due in part to the continual emergence of new viral variants and to synergistic interactions with other viruses and bacteria. There is a lack of understanding about how host responses work to control the infection and how other pathogens capitalize on the altered immune state. The complexity of multi‐pathogen infections makes dissecting contributing mechanisms, which may be non‐linear and occur on different time scales, challenging. Fortunately, mathematical models have been able to uncover infection control mechanisms, establish regulatory feedbacks, connect mechanisms across time scales, and determine the processes that dictate different disease outcomes. These models have tested existing hypotheses and generated new hypotheses, some of which have been subsequently tested and validated in the laboratory. They have been particularly a key in studying influenza‐bacteria coinfections and will be undoubtedly be useful in examining the interplay between influenza virus and other viruses. Here, I review recent advances in modeling influenza‐related infections, the novel biological insight that has been gained through modeling, the importance of model‐driven experimental design, and future directions of the field.

## 1 INTRODUCTION

Influenza viruses are important respiratory pathogens that infect 15‐65 million individuals each year in the United States with over 200 000 of these infections resulting in hospitalizations.^1^, ^2^ Despite available vaccines and antiviral therapies, influenza viruses remain a public health threat because they continue to evolve and novel strains emerge from zoonotic sources several times a century to cause pandemics. In addition, other viral or bacterial pathogens can invade and exacerbate influenza disease severity. Two or more pathogens can interact in ways that are not intuitive with numerous alterations occurring on varying time scales. Furthermore, different viral and/or bacterial strains, initial doses, timings of the secondary insult, and host immune status can result in distinct infection kinetics and disease outcomes.^3^, ^4^, ^5^, ^6^, ^7^, ^8^, ^9^, ^10^, ^11^ Examining every scenario and detailing different regulatory mechanisms is challenging even with animal models that can recapitulate many aspects of clinical diseases. This has limited our global understanding of influenza‐related diseases and emphasized a need for quantitative analyses that can detail the biology and evaluate different mechanisms simultaneously and rigorously.

During the past decade, mathematical models that describe host–pathogen and pathogen–pathogen interplay during influenza have made it possible to dissect critical mechanisms that drive the infection. The models have successfully quantified and predicted the viral load kinetics from clinical and experimental infections,^12^, ^13^, ^14^, ^15^, ^16^, ^17^, ^18^, ^19^, ^20^, ^21^, ^22^, ^23^, ^24^, ^25^ the symptoms that arise during infection,^12^, ^13^ the dynamics and efficiency of different host immune responses,^14^, ^17^, ^18^, ^25^, ^26^, ^27^, ^28^, ^29^, ^30^, ^31^, ^32^, ^33^, ^34^ the effect of different viral and host factors,^15^, ^16^, ^20^, ^21^, ^22^, ^35^ the efficacy and design of vaccines and antiviral therapies,^14^, ^19^, ^20^, ^21^, ^22^, ^36^, ^37^, ^38^, ^39^, ^40^ and the mechanisms of coinfection between influenza viruses and other viruses or bacteria.^41^, ^42^, ^43^, ^44^, ^45^

**Figure 1:**
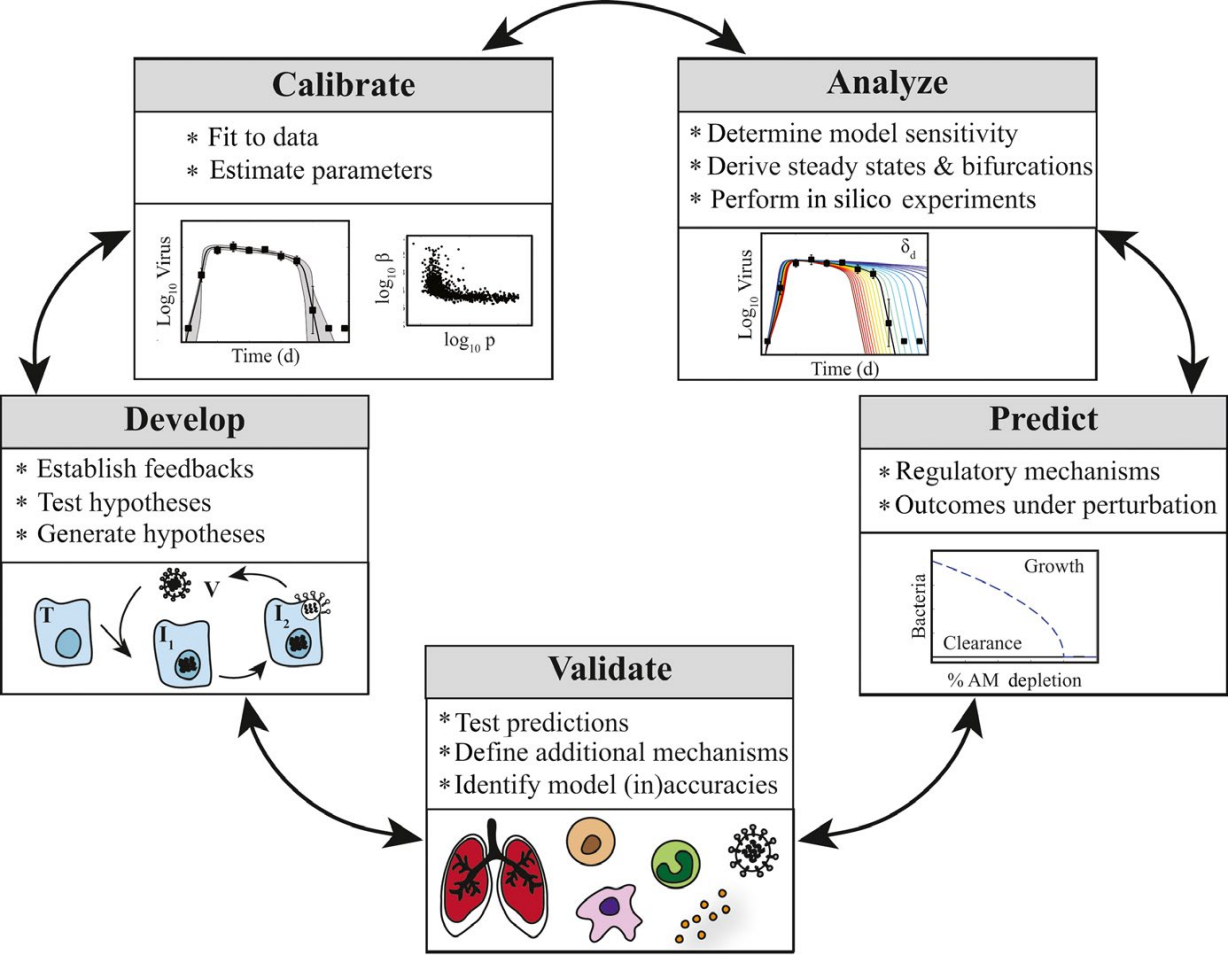
Data‐Driven Mathematical Modeling and Model‐Driven Experimental Design. Data‐driven mathematical modeling studies are iterative and entail developing a model to describe the underlying biology, calibrating the model to experimental or clinical data, analyzing the model with mathematical techniques, using the model to make predictions and design experiments, and validating the predictions in the laboratory or clinic

Remarkably, influenza viral load dynamics can be described using as few as 3‐4 equations for populations of uninfected cells, infected cells, and virus.^14^ The kinetics of host immune responses and/or coinfection with other pathogens can be accurately described by adding only 1‐2 more equations.^41^ Simple models like these are optimal because they readily allow for mathematical and statistical analyses that extract information about the underlying biology. Although models are typically first calibrated to data to ensure a robust recapitulation of the infection kinetics and to estimate the rates of growth and decay, this is not the only goal. The underlying model structure (eg, non‐linear feedbacks between different cell populations), the behavior of the resulting parameter estimates (eg, when two parameters are correlated), and in silico experiments that predict the response under perturbation (eg, with antivirals) can all reveal hidden regulatory mechanisms that may not be readily apparent from the data itself and/or cannot be tested in the clinic or laboratory. A schematic of this model‐experiment exchange is shown in Figure 1.

Improvements in the availability of quantitative data in recent years has led to more robust models being developed and to the predictions of some of these models being validated in the laboratory. One collection of studies, which are described here, illuminate the accuracy and predictive capability of mathematical models and the importance of designing confirmatory experiments to define new biology and improve the models. Here, I review current approaches in modeling influenza virus kinetics and host‐pathogen interplay, recent advances in modeling viral–bacterial and viral–viral coinfections, the techniques used to identify controlling mechanisms, biological interpretations of the model results, and the benefits of model‐driven experimental design.

## 2 MODELING INFLUENZA VIRUS INFECTIONS: THE GOLD STANDARD

Influenza A viruses infect the upper and lower respiratory tracts to cause acute, self‐limiting infections. The dynamics of the infection are rapid with the virus establishing quickly and replicating exponentially to high titers within 1‐2 days. In the majority of cases, the infection resolves within 7‐10 days, but viral loads can remain elevated in children and immunocompromised individuals. The mechanisms that drive these kinetics and how they might be altered by therapy or other pathogens are not well understood even though many of the contributory cytokines, chemokines, and cells are known.

Mathematical models have accurately described viral load kinetics without including equations for specific host responses.^46^, ^47^, ^48^ The models assume that susceptible epithelial cells (“target cells”) are limited and that virus declines once the majority of cells are infected.^14^ Accurate predictions have been made under this assumption, which does not specify the mechanisms by which target cells are limited. Nevertheless, several studies have challenged whether the approximation is accurate and how it relates to different host responses, such as type I interferons (IFN‐α and IFN‐β)^17^, ^25^ (A.M. Smith, unpublished data). Some studies have attempted to establish a comprehensive view of the host response^15^, ^35^, ^49^, ^50^ while others have taken a more focused approach.^14^, ^17^, ^26^, ^28^, ^34^ One benefit of models with reduced complexity is the availability of analytical tools that can facilitate a robust interpretation of the dynamics.

Until recently, progress in the field was plagued by a lack of sufficient data to parameterize/calibrate mathematical models, particularly those that included arms of the immune response.^46^ While viral load data remains the most prevalent type of data available, various immune factors have been measured on frequent enough time scales to be utilized in modeling studies, 15, 18, 51, 52(A.M. Smith, unpublished data). With these data, even the larger, more comprehensive models can be calibrated to data.^15^, ^35^, ^49^ In addition, efforts to improve parameter estimation algorithms and employ analytical techniques have significantly advanced our ability to generate robust predictions about the underlying biology.^16^, ^24^, ^49^, ^53^ Model results are now undergoing rigorous testing in the laboratory, which has confirmed their predictive capability and importance in identifying regulatory mechanisms driving influenza virus infections.

### 2.1 Viral kinetic model

**Figure 2:**
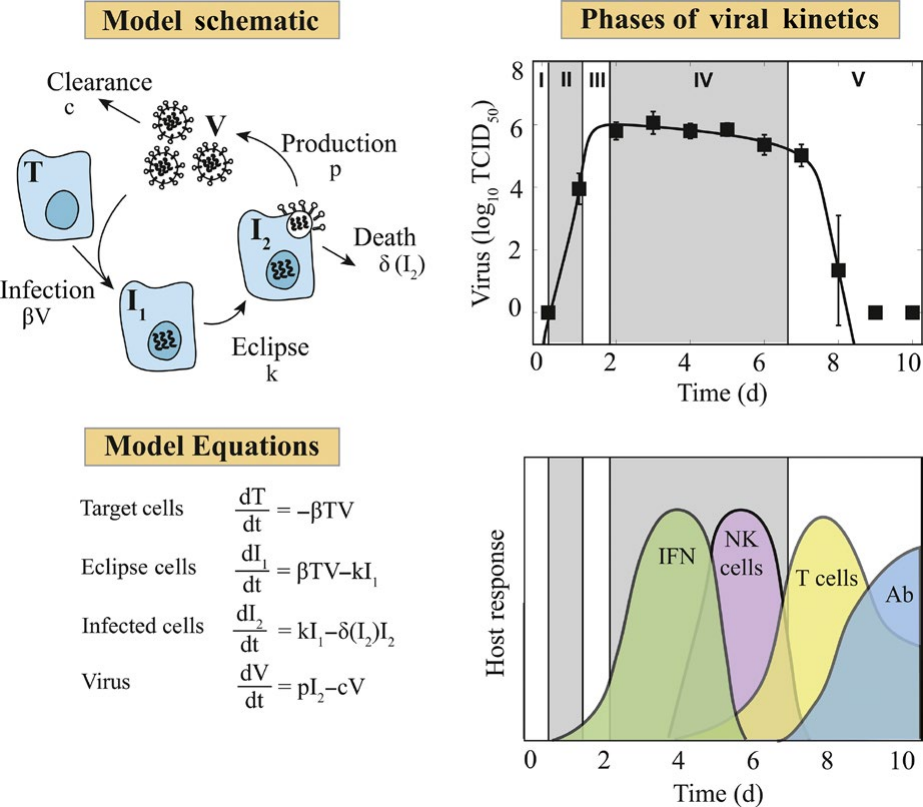
Viral Kinetic Model and Dynamics. A schematic of the standard viral kinetic model,^14^ associated equations, fit to data from mice infected with influenza A/Puerto Rico/34/8 (PR8),^24^ and timeline of major host responses are shown. The model tracks susceptible “target” cells (T), two classes of infected cells ($I_{1}$ and $I_{2}$), and virus (V). Target cells are infected by virus at rate βV. Once infected, the cells undergo an eclipse phase, which accounts for the time between infection and virus production. To account for these dynamics, infected cells are split into two classes, where k is the transition rate from unproductive to productive. Infected cells are lost at rate $\delta \left(I_{2}\right)$ per day. Virus is produced at rate p per infected cell and is cleared at rate c per day. The resulting model dynamics are shown for a saturating infected cell death rate, that is, $\delta \;\left(I_{2}\right)=\delta_{d}/\;\left(K_{\delta }+I_{2}\right)$, where $\delta_{d}/K_{\delta }$ is the maximum rate of clearance and $K_{\delta }$ is the half‐saturation constant. Viral kinetics generally split into ~5 phases: initial infection of cells, exponential growth, peak, a slow decay, and a fast decay/clearance. Major host responses influencing these phases include, type I interferons (IFN), natural killer (NK) cells, T cells, and antibody (Ab)

The majority of influenza virus infection models developed thus far have utilized a common model core, that is, the standard viral kinetic model^14^ (Figure 2). This model was first used to study influenza A virus (IAV) dynamics in humans. The model tracks susceptible “target” cells, infected cells not yet producing virus (ie, cells in the eclipse phase), infected cells producing virus, and free virus. The model schematic, equations, description, and fit to murine viral load data are shown in Figure 2. No specific immune dynamics are included in this model, but the rates of virus clearance and infected cell clearance (*c* and δ, respectively) encompass numerous virus‐ and immune‐related processes, including loss of virus infectivity, phagocytosis of viruses or cells, apoptosis of cells, viral cytopathic effects, killing of infected cells by immune effectors, or loss of the infected state by non‐cytolytic effects.

### 2.2 Model interpretation and the accuracy of the target cell limited hypothesis

A central assumption of the viral kinetic model (Figure 2) is that the number of target cells is limited.^14^ This manifests in the model as virus growth slowing and peaking once the majority of the target cells are infected. The model does not define what limits the target cells, which could be due to a variety of host immune responses. The assumption could be interpreted as (i) all cells within the respiratory tract become infected, which is possible but not generally observed^17^, ^25^, ^54^ (A.M. Smith, unpublished data), or (ii) there is a pre‐defined number of cells that will become infected (ie, where the initial number of target cells, *T*_0_, essentially defines the final number of infected cells). The lack of complete destruction of the respiratory tract, suggests that virus spread is regulated by host defense mechanisms. However, omitting specific immune control in the viral dynamics model does not invalidate the target cell limitation hypothesis, but may lead to disparate parameter values. Regardless of the underlying mechanism, influenza models with or without target cell limitation match much of the available viral titer data.^46^, ^47^, ^48^ In addition, the predicted dynamics of the infected cells (I_*2*_) agree well with the spatial spread, as measured by histomorphometry, even when only ~50%‐60% of the lung becomes infected (A.M. Smith, unpublished data). However, another study utilized GFP‐reporter virus data,^55^ which can also be used to track infected cells, and demonstrated that the target cell limited model breaks down for low dose infections.^25^ Simply reducing the number of initial target cells (*T*_0_) was insufficient to replicate the dose‐dependent dynamics.^25^ This may indicate a deficiency in the model or that some host responses are more functional with low dose infection, which has been proposed in other studies using low doses.^41^

### 2.3 Quantifying the rates of infection and the response to perturbation

Understanding time‐dependent mechanisms that control viral infection dynamics requires that mathematical models be calibrated to experimental or clinical data and thoroughly analyzed. Fitting a model to data ensures that the equations accurately describe the infection dynamics and provides estimates of the rates of infection, production, and clearance. It also begins to reveal the relationship between these rates and the strength needed to induce a change in the dynamics (eg, with drug therapy or coinfection). Further investigating how changing the rates affects outcome, for example, through sensitivity analysis, has generated predictions about the response to therapy^14^, ^19^, ^20^, ^21^, ^36^, ^56^ or coinfection with other pathogens.^41^, ^42^, ^43^, ^45^ Collectively, these types of analyses reveal aspects of influenza biology that are not immediately available from the experimental or clinical data alone.

With appropriate parameter estimation techniques, defining accurate and meaningful parameter values is possible. During model fitting, the log_10_ infectious viral load, which is typically in units of 50% tissue culture infectious dose (TCID_50_) or plaque forming units (PFU), is compared to the log_10_ output of the model. A variety of data fitting algorithms have been used, including adaptive simulated annealing (ASA),^24^ Monte Carlo Markov Chain (MCMC),^13^, ^15^, ^22^, ^35^, ^49^ Gaussian processes (GP),^53^ and maximum likelihood estimation (MLE).^16^, ^26^, ^41^ Until recently, it was relatively well accepted that the choice of estimation scheme is not critical. However, contrasting parameter estimates may result and some evidence suggests that ASA or GP methods can outperform MCMC and MLE methods in terms of accuracy, convergence, and run time.^24^, ^53^ Further investigation is needed to ensure robust results, particularly because MCMC methods are popular.

Uniquely identifying each parameter in a model has been challenging^57^, ^58^ but has not limited the predictive capability.^41^, ^44^ The standard viral kinetic model has seven unknown parameters (β, *p*,* k*,* c*, δ, *V*_*0*_, and *T*_*0*_ (see Figure 2)). In most studies, the values of the eclipse phase parameter (*k*) and initial target cells (*T*_*0*_) are fixed because their values can be calculated.^14^ However, these can be left free^24^, ^53^ without compromising the predictive capability. One problematic parameter has been the virus clearance rate (*c*), which often estimates to large values that may not be biologically relevant.^16^, ^24^ This is because the model attempts to capture the rapid decrease in free virus shortly after the infection is initiated as virus infects cells (~0‐4 hours). However, this challenge can be overcome by setting the initial free virus (*V*_*0*_) to zero (ie, *V*_*0 *_= 0) and assuming that the initial number of infected cells is positive (ie, *I*_*1 *_> 0).^24^ Using this assumption recovers virus clearance rates (*c*) that are more reliable.^24^

Ensuring robust predictions requires more than estimation of the model parameters. A thorough investigation into the uncertainty of the estimates and the corresponding model solution is also required. This has been particularly true when attempting to determine significant differences in parameter estimates generated by fitting a model to data obtained under varied experimental conditions, such as during infection with different virus strains,^16^, ^41^ with different doses,^15^, ^59^ in different host genetic backgrounds,^60^ or in different aged individuals.^59^, ^61^ For this, ensemble‐style methods have been particularly useful. Plotting the resulting parameters within a 95% confidence interval (CI) as histograms and as two‐dimensional (2D) or 3D projections of the parameter space is critical to effective interpretation of the model results. Unsurprisingly, parameters are often correlated (eg, virus infection and production,^17^ virus production and clearance,^24^ or virus clearance and infected cell clearance 16), which suggests that the data is insufficient to distinguish between these processes. For example, similar viral load dynamics may be possible with slow virus growth and clearance and with fast virus production and clearance. If the goal was to distinguish between these possibilities, additional data would be necessary. Utilizing multi‐variable data, such as infectious virus and viral RNA copies, can reduce uncertainty,^62^ but this comes at the expense of increasing the number of parameters and equations. Importantly, correlated parameters do not inhibit the accuracy of the parameter estimates or the insight gained from the model.^41^, ^44^ Knowledge about correlated parameters should not encourage fixing parameters or fitting combinations of parameters because this may inadvertently skew the results and lead to important information being lost.

Following model calibration (ie, fit to data), in silico experiments are used to predict how the dynamics shift in response to different stimuli (eg, antiviral therapy or infection with another virus or bacteria). The effects that different parameters have on the model dynamics can be observed by simulating the model equations and increasing or decreasing the parameter values of interest. This is known as a sensitivity analysis. Perturbing more sensitive processes results in larger downstream effects compared to changes in less sensitive processes. Comparing the results of this analysis to data where experimental conditions are altered, such as data from knockout animals, can be challenging. The inability of some models to predict the response in perturbed conditions^63^ may be due to the simplicity of the model or a misinterpretation of the data. For dose‐dependent kinetics, including features of the host response can improve accuracy and predictive power.^26^, ^64^. However, data from knockout animals, for example, may not reflect a change in a single variable and other immune factors may be affected and contribute to the dynamical differences observed in the infection data. Simultaneous measurement of other variables (ie, immune cells and cytokines) is likely required to evaluate whether other populations are skewed. Further understanding of the model limitations should then naturally arise.

### 2.4 Insight from analytical solutions: time‐dependent mechanisms

The predictive capability of influenza models goes beyond data fitting, parameter estimation, and sensitivity analysis. The simplicity of the model is beneficial because additional mathematical analyses are feasible.^65^ It can be easily observed that viral load dynamics split into two log‐linear (ie, exponential) phases: growth and decay. During the initial growth period, few target cells are infected and their population remains relatively constant (A.M. Smith, unpublished data). This information was used to obtain an equation that describes exponential virus growth^65^: $V_{1}\left(t\right)=\alpha_{1}e^{\lambda t}$, where $\lambda \approx {\left(\beta pkT_{0}\right)}^{1/3}-\left(k+c+\delta \right)/3$) is the slope of the viral growth and α_1_ is a constant.^23^, ^65^ All of the model parameters describing the processes of infection and clearance (ie, virus infection (*β*), production (*p*), and clearance (*c*), eclipse phase (*k*), and infected cell clearance (δ)) have a role in determining the speed of virus expansion. This solution matches the viral load kinetics during the first ~2 days of the infection.^16^, ^65^ The point where virus growth slows (ie, where $V_{1}\left(t\right)$ deviates from the numerical solution of the model occurs at ~2 days post‐infection (pi). This signifies the end of the exponential growth phase and the point where antivirals that target the viral life‐cycle (eg, neuraminidase (NA) inhibitors (NAIs) or matrix‐2 inhibitors (M2Is)) begin losing efficacy (discussed further below). The prediction agrees with clinical and laboratory observations that antivirals are not effective when given after 48 hours of symptom onset^66^ and provides an explanation for the differential efficacy of antivirals against influenza viruses.

Prior to virus decay, there is a short, non‐linear period (~12 hours) between virus growth and decay where the growth slows prior to the peak.^65^ During the resolution period, most available cells have become infected and there are few target cells remaining (*T *≈ 0). This information was used to obtain an equation that describes exponential virus decay^65^: $V_{2}\left(t\right)=\alpha_{2}e^{-\delta t}+\alpha_{3}e^{-ct}+\alpha_{4}e^{-kt}$, where the α_i_s are constants. This solution is less complex than $V_{1}\left(t\right)$ and defines the peak and infection resolution. Here, the peak shape is dictated by the rates of eclipse transition (*k*), virus clearance (*c*), and infected cell clearance (δ). After the peak, the infected cell death rate (*δ*) controls the rate of decay (ie, $V_{2}\left(t\right)\approx V_{p}e^{-\delta t}$, where $V_{p}$ is the peak viral load). Having solutions like these that detail the time‐dependent contribution of each infection process to the viral dynamics has been beneficial in establishing robust interpretations of the data and models.

## 3 DETAILING IMMUNE CONTROL DURING INFLUENZA VIRUS INFECTION

Throughout influenza virus infection, various immune responses are employed to limit virus spread and maintain integrity of the epithelium (Figure 2).^67^ Interferons, including IFN‐β (type I), IFN‐λ (type III), and to a lesser extent IFN‐α (type I), are produced early in the infection. These are most prevalent in the lung from ~2 to 5 days pi and coincide with increases in neutrophils, natural killer (NK) cells, and pro‐inflammatory cytokines. Subsequently, T cells and B cells become activated and infiltrate the infected area. Although the standard viral dynamics model can replicate viral load data from a variety of systems and generate accurate predictions without including these dynamics, recent studies have noted some insufficiencies.^17^, ^24^, ^25^, ^34^, ^36^ Some viral load data do not follow the classical log‐linear viral dynamics behavior and exhibit either a two‐phased decay and/or a second, smaller peak (eg, as in 14, 24, 52, 68, 69 and references therein). Although complex immunological models have been used to explain these features,^15^, ^17^, ^18^, ^26^, ^27^, ^28^, ^34^, ^35^, ^49^ data on specific immune components is often lacking. Fortunately, adding only one parameter to the standard viral kinetic model to induce a non‐linearity (ie, saturation) in the rate of infected cell clearance is sufficient to switch the dynamics from a monophasic decay to a biphasic decay (Figure 2).^24^, ^70^, ^71^ That is, the rate of infected cell clearance decreases as the number of these cells increases. A saturating infected cell clearance rate may reflect a switch from innate to adaptive control, a “handling time” (eg, the time taken for a T cell to remove an infected cell), and/or cell activation (eg, macrophage (MΦ), T cell, or B cell). How and why the rate changes remain open questions, but it is likely connected to the processes driving the rate of T‐cell expansion (A.M. Smith, unpublished data). A plateau of viral loads can be reproduced in other ways, for example, by including equations for specific immune components.^14^, ^34^

Mechanistic host response models have been built to examine the activation and production of cells or cytokines and the efficacy of different factors (ie, cells or antibodies) in removing virus or infected cells.^15^, ^18^, ^27^, ^28^, ^29^, ^30^, ^31^, ^32^, ^33^, ^34^, ^35^, ^49^, ^50^, ^72^ The models range in complexity with some attempting to incorporate several pro‐inflammatory cytokines, anti‐inflammatory cytokines, and cell populations.^15^, ^35^, ^49^, ^50^ The most common responses modeled are type I IFNs, CD8^+^ T cells, and antibodies because of their profound influence during influenza virus infection. Some studies have used generalized equations to reflect the functions of other cytokines or cell types, which is particularly beneficial when the dynamics of specific responses are unknown.^28^, ^31^, ^32^ Immune control is typically incorporated through use of different functional forms for the virus clearance (c) and the infected cell clearance (δ), for example, $dI/dt=kI_{1}-\delta_{1}\;\left(X\right)I_{2}-\delta_{2}\;\left(Y\right)I_{2}$ and $dV/dt=pI_{2}-c\;\left(Z\right)V$. Here, infected cell clearance is a function of innate mechanisms ($\delta_{1}\left(X\right)$), where *X* could denote MΦs, neutrophils, and/or NK cells, and adaptive mechanisms ($\delta_{2}\left(Y\right)$), where *Y* denotes CD8^+^ T cells. Similarly, virus clearance is a function of different mechanisms (c(Z)), where *Z* could denote antibodies (Ab) and/or MΦ. In addition to these functions, other rates may be affected (eg, virus production via IFN) and equations for the immune component of interest are included. The discussion here focuses on the two most modeled immune factors: type I IFNs and CD8^+^ T cells.

### 3.1 The antiviral type I interferon response

The type I IFN response has potent antiviral activity and is important for control of influenza virus infections.^67^ Type I IFN gene transcripts are upregulated within 24 hours after infection,^51^ which leads to the production of IFN‐α and IFN‐β. IFN‐α and IFN‐β are first observed 48 hours pi with continued production until ~5 days pi for IFN‐β and until after 10 days pi for IFN‐α^52^ (A.M. Smith, unpublished data). They work to reduce the rate of virus production and spread of the infection.^67^, ^73^, ^74^ In addition, these type I IFNs promote the local inflammatory response 75, 76, 77, 78, 79and IFN‐α has anti‐inflammatory properties.^80^, ^81^ However, influenza viruses can antagonize the IFN response within infected epithelial cells, which is primarily mediated by its non‐structural protein, NS1.^82^, ^83^

The majority of models developed thus far have focused on the effect of IFN (*F*) in limiting virus production from infected cells: $dV/dt=\left(p/1+\varepsilon_{F}F\right)I_{2}-cV$, where $\varepsilon_{F}$ is the efficiency of IFN in reducing virus production.^14^ Time delays have been included in some models to account for the delayed detection of type I IFNs.^14^ It is unclear if this is a delay in production, a lack of assay sensitivity, or to other dynamics (eg, uptake of IFN into cells). Including IFN within the model either by reducing the rate of virus production ($\varepsilon_{F}$) or reducing the number of target cells (ie, cell refraction; dT/dt=−βTV−σFT, where σF is the rate of targets cells entering a refractory state) limits virus growth. Some studies also include reversion of cells from the refractory state.^27^, ^34^, ^72^ However, in vitro studies suggest that IFN‐induced cell refraction is long‐lived, so inclusion of this term may not be supported biologically.^84^

IFN‐α and IFN‐β are most abundant during mid‐infection (~2‐5 days pi) when viral loads are relatively constant. In addition, the first detectable IFN is after the time when virus has peaked. Thus, directly connecting IFN related effects to virus suppression is difficult using only viral load measurements. This could mean that other host response mechanisms are more potent in slowing virus growth. Emerging techniques that track the infected cell dynamics may help reconcile these difficulties^55^(A.M. Smith, unpublished data). These data indicate that there are relatively few infected cells early in the infection, which is when virus is most rapidly increasing, and that the number of new infections increases most profoundly during mid‐infection when viral loads are constant and type I IFNs are most abundant (A.M. Smith, unpublished data). While it remains difficult to directly connect these dynamics, the data are provocative. New models investigating IFN heterogeneity and viral antagonism may help interpret the data.^85^ However, we must reconcile data from some IFN perturbation experiments that suggest viral loads are altered only in the later stages of infection when IFN is absent.^63^ These data may indicate that IFN has more potent effects (eg, on inflammation) other than limiting virus infection of target cells.

Type I IFNs do aid in the recruitment of inflammatory cells and improve efficacy of the adaptive immune response (eg, T cells).^75^, ^76^, ^77^, ^78^, ^79^ Understanding the dynamics of IFN‐producing cells and their relative contribution to the total amount of IFN may be required. However, modeling specific cell populations, even with experimental data, may inadvertently bias model parameter estimates and/or model predictions because these cells change on varying time scales and have inherently heterogeneous cytokine production. The idea that IFNs influence the recruitment and efficacy of the cellular immune response has been modeled with the equation $dI_{2}/dt=kI_{1}-\delta I_{2}-\kappa FI_{2}$, where κF is the rate of IFN‐induced infected cell clearance.^34^ This was assumed to reflect infected cell removal by NK cells, which enter earlier than CD8 T cells, and still required use of a piecewise exponential function for the adaptive response.^34^ Thus, it was not sufficiently mechanistic to assess the impact of IFNs on CD8^+^ T‐cell efficiency. No study has assessed the anti‐inflammatory effects of IFN‐α. More work is clearly needed to tease apart the effects of IFN during IAV infection. In addition, because type I IFNs are important mediators of viral–bacterial coinfection severity^86^, ^87^, ^88^, ^89^, ^90^ and likely have a role in viral–viral coinfection dynamics, building new IFN models that are calibrated to data are pivotal.

### 3.2 CD8^+^ T cell‐mediated virus control and waning immunity

CD8^+^ T cells are responsible for clearing virus infected cells and resolving the infection.^51^, ^91^, ^92^ The infiltration of these cells into the respiratory tract is concurrent with rapid virus decay and the conclusion of the infection. The most abundant gene transcripts during the later stages of viral clearance are ones involved in T‐cell activation and induction of apoptosis.^51^ Models describing the CD8^+^ T‐cell response have investigated their differentiation, proliferation, specificity, efficacy in killing infected cells, and how they can be manipulated to provide long‐lived protection from natural infection or by vaccination.^18^, ^26^, ^27^, ^28^, ^32^, ^72^ Although the published models have different formulations, the resulting dynamics from each can successfully fit pulmonary CD8^+^ T cell counts from both humans and mice. In addition, the models robustly predict that resolution accelerates as the number of CD8^+^ T cells increases, that viral clearance is sensitive to the rate of T‐cell expansion, and that T‐cell efficiency increases with density (A.M. Smith, unpublished data). Interestingly, the latter finding reflects dynamics similar to the viral kinetic model with biphasic decay (Figure 2), which excludes T cell‐mediated clearance.^24^ Assessing how the non‐linearity in the rate of infected cell clearance relates to granzyme B production and other host responses like type I IFNs has yet to be modeled but may provide further insight into the T‐cell response.

Dynamical models for CD8^+^ T‐cell control of influenza virus infection have also yielded important information about long‐term protective immunity.^27^, ^28^, ^72^ One study predicted that repeated exposure to influenza viruses promotes the plateauing of memory CD8^+^ T cells and that immediate protection from subsequent insults may be lost because memory cells residing in the lung decay after each infection.^28^ This may help explain why individuals experience multiple infections in their lifetime. Moreover, infections with different pathogens species can affect the number of T cells and may lead to repeated influenza virus infections. For example, CD4^+^ and CD8^+^ T cells decrease significantly during influenza coinfection with bacteria.^93^ How this impacts viral clearances and shapes later responses is unknown. These interactions have important implications for infections in the elderly, who are more prone to developing pneumonia. More data on the longevity of resident T cells and how infection history influences their dynamics is necessary to address these questions.

## 4 VIRAL–VIRAL COINFECTION KINETICS

Respiratory viruses like influenza virus, respiratory syncytial virus (RSV), parainfluenza virus (PIV), and rhinovirus (RV) are easily transmitted and have overlapping seasons. Thus, it is not surprising that multiple viruses can be detected within infected individuals.^94^, ^95^, ^96^, ^97^, ^98^, ^99^, ^100^, ^101^ The specific outcomes that result from multi‐virus infections and the underlying mechanisms that drive their interactions are not well understood. There is evidence that virus interactions can be either synergistic or inhibitory because they often infect the same cell types and initiate similar inflammatory pathways.^11^ Some responses (eg, IFN) may have dynamics that are virus‐specific,^102^ and the resulting interactions depend on the pathogen strain, dose, and order. For example, PIVs can increase the rate of IAV growth by fusing cells together and facilitating cell‐to‐cell spread.^103^ This occurs without any noticeable effect on PIV replication. In a similar interaction, IAV infection attenuates RSV by inhibiting protein synthesis and does so with little impact on IAV titers.^104^ In contrast, pre‐infection with RSV or RV does not impact influenza virus replication but can reduce disease severity.^105^, ^106^ For RV, this is due to enhanced clearance of influenza virions.^105^ Unlike IAV‐RSV coinfection, superinfection with RV enhances influenza disease severity.^105^

**Figure 3:**
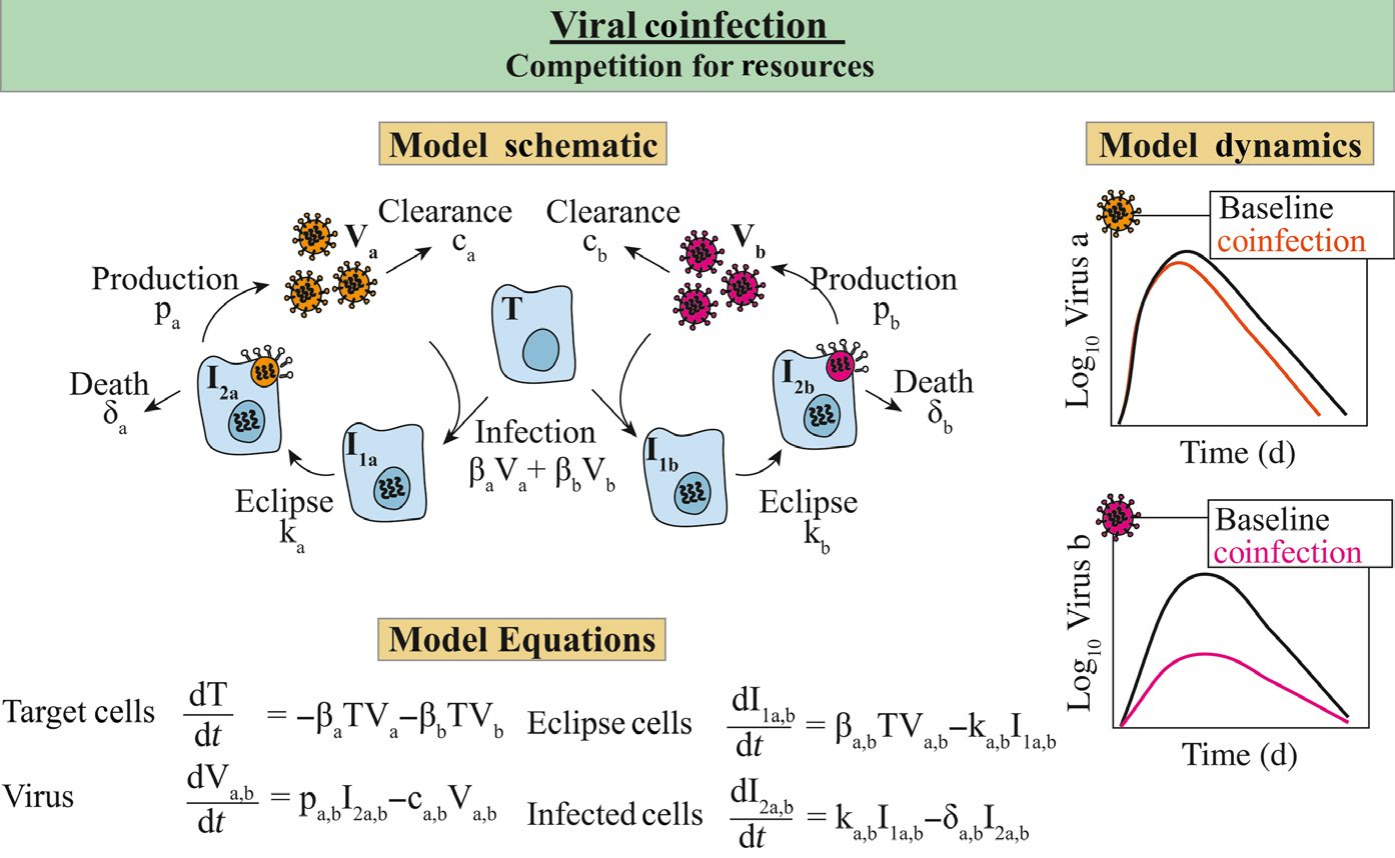
Viral–Viral Coinfection Model. Model schematic, equations, and dynamics for a viral–viral coinfection where two viruses (virus‐a and virus‐b) compete for target cells.^42^ In this model, target cells can be infected by virus‐a (orange) with rate $\beta_{a}$ and by virus‐b (magenta) with rate $\beta_{b}$. The model structure from the standard viral kinetic model is retained, but different rates of the eclipse phase ($k_{a,b}$), infected cell clearance ($\delta_{a,b}$), virus production ($p_{a,b}$), and virus clearance ($c_{a,b}$) are allowed. This interaction results in significantly reduced viral loads for the slower growing virus (magenta) and negligible declines in viral loads for the faster growing virus (orange). See Ref. 42 for fits to viral load data

Only recently was a mathematical model developed to begin examining respiratory virus coinfections.^42^ The model assessed how resource competition between two viruses could alter viral load dynamics of each virus (Figure 3). In this model, target cells could be infected by either virus, which have different infection rates (ie, $\beta_{a}$ and $\beta_{b}$) (see Figure 3). The remaining populations retained the structure of the standard viral kinetic model and allowed for different rates of the eclipse phase ($k_{a,b}$), infected cell clearance ($\delta_{a,b}$), virus production ($p_{a,b}$), and virus clearance ($c_{a,b}$). Coinfection of single cells was excluded. The model replicated in vitro data from coinfection with IAV and RSV, where IAV inhibits RSV growth,^104^ and with IAV and PIV, where PIV enhances IAV growth.^103^ A key result was that varied infection kinetics and outcomes could manifest from changing the virus dose or the intrinsic virus growth rate. Although RSV dose may not affect the interaction during IAV‐RSV coinfection,^106^ the finding is relevant for RV‐IAV coinfection.^105^ However, interference in the infection of epithelial cells is not the proposed mechanism for these viruses.^105^ The model prediction could be interpreted in another way. That is, when the interaction between viruses is competitive, target cells become limited because the first or fastest virus infects the majority of these cells and, thus, limits the second virus. The reduction in target cells could reflect other mechanisms, such as changes in type I IFNs or macrophages. Although few studies have modeled viral–viral coinfection, new information about the underlying biology should arise as more experimental data emerges and new models are developed.

## 5 INFLUENZA‐BACTERIA COINFECTION KINETICS

Complications arising from bacterial superinfections have accounted for a significant percentage of influenza‐related morbidity and mortality during pandemic influenza (40%‐95%) 107, 108, 109, 110 and during seasonal influenza (2%‐35%).^111^ Common pathogens responsible for this enhanced disease include the gram‐positive bacteria *Streptococcus pneumoniae* (pneumococcus) and *Staphylococcus aureus*.^107^ Similar to some virus–virus pairings, pre‐infection with bacteria can limit influenza virus infection.^112^ Antecedent bacterial infections prior to influenza have not been well studied. However, a wealth of knowledge exists about the viral, bacterial, and host responses that affect bacterial invasion and the development of pneumonia in influenza‐infected hosts.^3^, ^4^, ^5^, ^6^, ^7^, ^8^, ^9^, ^10^ Multiple studies indicate immune exacerbation as a key driver of coinfection severity. A plethora of immune responses, including MΦs, neutrophils, NK cells, T cells, B cells, and various cytokines and chemokines, are altered during influenza virus infection and/or during bacterial coinfection.^3^, ^4^, ^5^, ^6^, ^7^, ^8^, ^9^, ^10^ The varying time scales and interconnectedness of host responses has made establishing the contribution and regulation of each factor complicated. In recent years, a series of iterative mathematical and experimental studies unraveled some of the complex host‐pathogen interactions and identified important mechanisms that drive bacterial establishment during influenza virus infection (discussed below).^41^, ^44^, ^113^ Examining the host‐pathogen feedbacks during influenza‐bacterial coinfection first required a quantitative description of a pneumococcal infection.^64^

### 5.1 Host control of pneumococcal pneumonia

Pneumococci readily colonize the nasopharynx of healthy adults and children^114^, ^115^, ^116^ and occasionally migrate to other tissues to cause severe disease, such as otitis media, pneumonia, meningitis, and septicemia.^117^ When pneumococci invade the lung, host responses are relatively efficient in clearing the bacteria. If pathogen removal mechanisms like the ciliated epithelium or MΦs become compromised, such as from comorbidities like an underlying respiratory disease or virus infection, bacteria can permeate the lower airways. Infection with more virulent pneumococcal strains and/or a high dose can also result in pneumonia.^118^

For most bacterial infections, a simple model like the ones used for viruses cannot be used. This is because pneumococci are extracellular pathogens and their growth and clearance dynamics are highly dependent on interactions with host immune responses.^60^, ^64^ Indeed, modeling pneumococcal dynamics required equations for several arms of the immune response to accurately capture bacterial kinetics from infections with varied initial doses.^64^ Fortunately, many of the important players, including alveolar macrophages (aMΦs), neutrophils, inflammatory MΦ (iMΦ), and pro‐inflammatory cytokines, were known. However, the regulatory feedbacks between these populations had not been established. This presents one of the main challenges but also a major benefit to modeling infection kinetics. The model I developed with coinvestigators described the interplay between pneumococci, aMΦs, neutrophils, iMΦs, cytokine signaling between these populations, and the resulting inflammation/damage caused by bacterial‐mediated injury of healthy epithelial cells and by neutrophil infiltration and cytotoxicity.^64^

This model mimics infection data from a variety of conditions, including changes in bacterial dose, bacterial strain, murine strain, and under antibacterial therapy.^60^, ^64^, ^119^ The model accurately predicts that the ratio of aMΦs to bacteria regulates bacterial growth in the early stages of infection and that there is a critical threshold for which a clearance phenotype can be attained.^64^ Indeed, this has been observed in several data sets 44, 120, 121, 122 and recently shown for varying combinations of aMΦs and bacteria.^44^ The subsequent neutrophil response further dictates bacterial growth kinetics and outcome.^60^, ^64^, ^119^ Sensitivity of the system revealed that neutrophil‐mediated damage of the epithelium is an important predictor of outcome.^64^ Understanding the role of tissue damage during infections is important and often more closely related to the probability of survival than to pathogen levels. Modeling immune‐mediated lung damage has not been attempted for influenza but will undoubtedly prove useful, particularly because tissue damage and defects in tissue repair affect influenza‐bacteria related mortality.^123^

### 5.2 Host‐pathogen regulation during influenza‐pneumococcal coinfection

Throughout influenza virus infection, epithelial cells are infected and die, and inflammation accumulates as host immune responses work to halt virus spread. As lung tissue becomes injured and the host immune response weakens, bacterial pathogens readily invade and cause pneumonia.^3^, ^4^, ^6^, ^7^, ^8^, ^9^, ^10^, ^124^ Heightened lethality occurs when bacteria invade during the virus resolution phase with the maximum synergistic effect at 7 days post‐influenza.^112^

**Figure 4:**
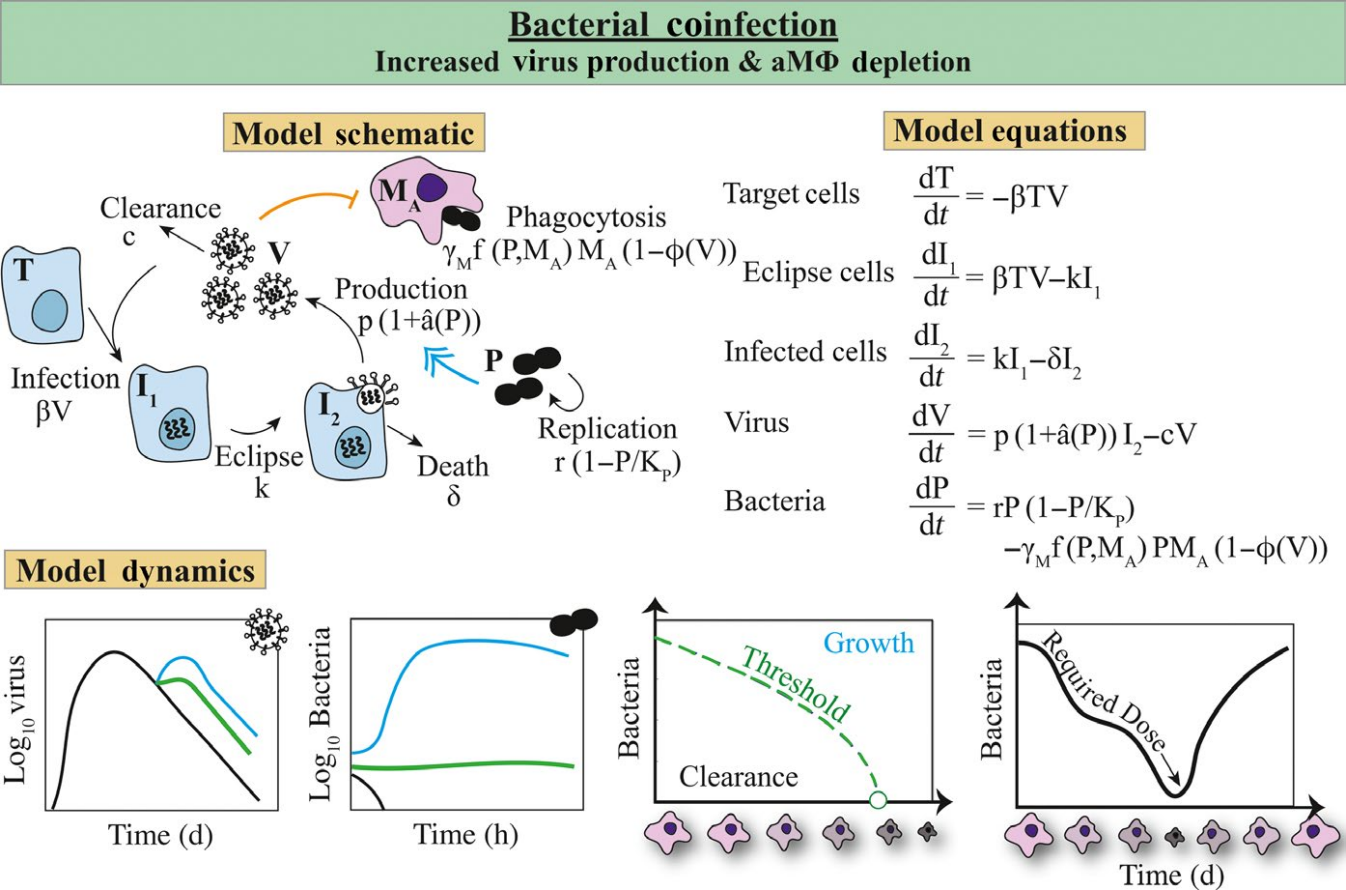
Viral–Bacterial Coinfection Model. Model schematic, equations, and dynamics for a viral–bacterial coinfection model where influenza virus depletes aMΦ ($M_{A}$) or renders them dysfunctional according to $\hat{\phi }\left(V\right)$, which reduces bacterial clearance.^41^ In addition, bacteria (P) enhances virus production according to the function *â(P)*. Bacteria replicate logistically ($r(1-P/K_{P})$) and are cleared at rate $\gamma_{M}f\left(P,M_{A}\right)M_{A}$. The remaining equations are given by the standard viral kinetic model. These interactions result in a rebound of virus and rapid bacterial growth (cyan). The bacterial growth trajectory is defined by a threshold (green),^44^ such that bacterial titers will decline when bacteria‐aMΦ pairs are below the threshold (black), remain constant when bacteria‐aMΦ pairs are at the threshold (green), and increase when bacteria‐aMΦ pairs are above the threshold (cyan). Because aMΦs decline throughout an influenza virus infection, the dose required to initiate an infection also declines. See Ref. 41 for fits to viral and bacterial load data, Refs. 113, 125, 126 for validation of the model predictions, and Ref. 44 for validation of the threshold

Following bacterial infection, viral loads rebound and bacterial titers increase to high levels within ~24 hours (Figure 4).^41^ In addition, many host responses are elevated (eg, type I IFNs) while others are dampened (eg, T cells). To investigate the mechanisms that govern these dynamics and begin disentangling the host immune response, the standard viral kinetic model was paired with the aMΦ subset of the pneumococcal model (Figure 4).^41^ The remaining populations (ie, neutrophils, inflammatory macrophages, cytokines, and damage) in the pneumococcal model were not used because corresponding models that describe the dynamics of these populations during IAV infection are not available. The coinfection model altered different terms in the model to examine both pre‐defined hypotheses and novel hypotheses. The dynamics generated by the model are in good agreement with experimental data and showed that only two alterations were needed to explain the dynamics (Figure 4).^41^ In the model, bacteria increase the rate of virus production from infected epithelial cells ($pI_{2}$) according to the saturating function $\hat{a}\left(P\right)=aP^{z}$ (Figure 4). This term drives the viral rebound. There was no pre‐defined hypothesis or evidence for this increase, but its inclusion in the model was critical. This novel hypothesis subsequently guided several in vitro experimental studies,^125^, ^126^, ^127^ where at least two potential underlying mechanisms were discovered. First, *S. aureus*, another common coinfecting bacteria, was shown to inhibit IFN signaling in influenza‐infected cells, which resulted in increased virus production.^125^ Although it is unknown if pneumococci have this same ability and to what extent this occurs in vivo, particularly considering the enhanced IFN levels during coinfection,^86^, ^87^, ^88^ it is an intriguing finding and validates the model‐generated hypothesis. Second, pneumococcal neuraminidases, NanA and NanB, have been shown to promote virus replication 126, 128 presumably through cleavage of viral NA. Unsurprisingly, increased viral loads were not observed when the two pathogens were simultaneously administered to cell cultures.^127^ This reduced synergism is consistent with in vivo results indicating that the order and timing between pathogens is important.^112^

The model also predicted that virus infection decreases the rate of bacterial clearance by aMΦs according to the saturating function $\hat{\phi }\left(V\right)=\phi V/(K_{\mathrm{PV}}+V)$ , where $K_{\mathrm{PV}}$ is the half‐saturation constant (Figure 4). This term drives bacterial invasion and was initially included to assess previous reports that aMΦs became dysfunctional during influenza.^120^ Although the model could not distinguish whether these cells were functionally impaired or were lost during infection, the changes to the aMΦ population were sufficient to drive the bacterial load dynamics.^41^ In addition, the resulting parameter estimate indicated that the strength of this reduction was significant (ie, ϕ(V)=85−90%). A follow‐up experimental study that tracked the aMΦ population with a labeling dye and employed a novel and robust flow cytometry gating strategy better defined the aMΦ dynamics during IAV infection.^113^ This study showed a profound depletion of aMΦs over the course of influenza,^113^ which may be specific to BALB/cJ mice.^129^ In C57BL/6 mice, aMΦs may be functionally inhibited.^129^ Fortunately, the model remains accurate because the underlying mechanism is not defined by the model. Remarkably, the experimental data showed that aMΦs were reduced at 7 days post‐influenza by the exact value that the model predicted, that is, 85%‐90%.^113^ This study effectively validated the model and the estimate of $\hat{\phi }\left(V\right)$. In addition, the data and model together helped identify why bacterial invasion 7 days after influenza results in maximal lethality.^112^ How aMΦs become depleted and how their loss alters other host responses and lung function 76, 130, 131, 132 remain open questions.

Parameter estimation played a key role in identifying these mechanisms and in determining that they are independent.^41^ The lack of correlation between the parameters involved in the two functions, $\hat{a}\left(V\right)$ and $\hat{\phi }\left(V\right)$, suggested that they described distinct processes. Unsurprisingly, there were correlations within each function (ie, *a* is correlated to *z*, and ϕ is correlated to $K_{\mathrm{PV}}$).^41^ Notably, these correlations did not inhibit accurate parameter values from being obtained.^41^, ^113^ These studies illuminate the critical nature of validating a model's predictions to expand its capabilities through correcting any inaccuracies (eg, altering functional forms or adding new equations) and completing new analyses (eg, as in 36, 44). It remains unclear if the function describing the increase in virus production $(\hat{a}\left(P\right)=aP^{z}$) is accurate. However, the new aMΦ data suggested that the effect on these cells does not saturate (ie, $\hat{\phi }\left(V\right)\neq \phi V/\left(K_{\mathrm{PV}}+V\right)$).A more mechanistic model for aMΦ interactions with influenza virus is likely required. Nevertheless, approximating aMΦ depletion $\hat{\phi }\left(V\right)$ through produced robust predictions.^41^, ^44^

### 5.3 The non‐linear threshold regulating phenotype and heterogeneity

The new knowledge about aMΦ dynamics and the connection of these data to $\hat{\phi }\left(V\right)$ allowed for another iteration of the model‐experiment exchange.^44^ By simulating the model with values for $\hat{\phi }\left(V\right)$ between 0 (0% depletion) and 1 (100% depletion), it was observed that this parameter is a bifurcation parameter that regulates bacterial growth trajectories.^44^ Mathematical analyses were used to derive the non‐linear threshold that defines the dynamical switch between growth and clearance phenotypes (Figure 4). That is, bacteria‐aMΦ pairs that fall below the threshold will result in bacterial clearance while pairings above the threshold will lead to bacterial growth. The threshold can be used to identify the dose needed for successful bacterial invasion during influenza. It also suggests that there is a critical point where any dose will initiate the secondary infection (dot on threshold curve in Figure 4). This is defined by a relation between the rates of bacterial growth (*r*) and clearance ($\gamma_{M}M_{A}$),that is, ${\hat{\phi }}_{\mathrm{crit}}=1-r/\left(\gamma_{M}M_{A}\right)$. This information was used to design confirmatory experiments, which examined bacterial kinetics for over 20 different combinations of bacteria and aMΦs.^44^ The data showed that the threshold was accurate, the rate of bacterial growth/clearance increases with distance above/below the threshold, the phenotype switches if complete clearance is not attained within ~4 hours, and pairings below the threshold result in heterogeneous bacterial titers.^44^ This information suggests that the behavior can be predicted for any bacteria‐aMΦ pairing, which is ideal. It also aids in the interpretation of bacterial load data and allows for exploration of therapies that manipulate bacterial loads (eg, antibiotics) and aMΦs (eg, immunotherapy or antivirals).^36^

### 5.4 Defining the contribution of other mechanisms

In addition to identifying the mechanisms described above, the model also defined the time scales on which they act. For high‐dose infection, the slope in the bacterial dynamics changes at ~10 hours post‐bacterial infection.^41^ This indicates that the contribution of aMΦs to clearance is short lived, which has been observed experimentally.^44^, ^120^, ^121^, ^122^ However, bacteria grow exponentially after this time, which suggests that neutrophils have little contribution to controlling bacterial kinetics when the dose is sufficiently high.^41^ This is consistent with experimental evidence that these cells become dysfunctional throughout influenza.^133^, ^134^, ^135^, ^136^, ^137^ The contribution from neutrophils may be higher during low‐dose infection,^41^ but this has not been explored in detail. A better understanding of how other cell types and cytokines regulate pathogen kinetics and outcomes of influenza‐bacterial coinfection should manifest as new models for influenza are developed.

### 5.5 Connecting mathematically derived mechanisms to omics data

The focus of many infectious disease studies has recently switched from collecting qualitative data to collecting large, quantitative ‘omics’ data sets that simultaneously measures multiple variables (eg, proteins, metabolic factors, and viral and host transcripts). Omics studies require computational approaches that assess correlations between different measurements. The computational methods for this type of data are frequently network‐based and take into account known interactions (eg, protein–protein) or predicted interactions (ie, correlations) between biological variables. However, one limitation of this analysis is that it cannot readily assess the dynamic feedback of variables (eg, non‐linearities like saturating effects), which often occur on distinct time scales. In contrast, mathematical descriptions of infection processes quantitate the intricate host‐pathogen feedbacks and link causation and correlation. Kinetic models determine the time scales of various mechanisms, the rate, magnitude, and effectiveness of immune responses, and whether bifurcating behavior is possible. As more omics data become available, it will be valuable to relate this information to the network analyses because each approach may have related and distinct conclusions. For instance, an omics study that profiled gene expression patterns during influenza‐pneumococcal coinfection found that lethality is correlated with an early increase in bacterial replication.^123^ Interestingly, the kinetic studies described above made the same conclusion but also identified the regulatory mechanism that governs this behavior.^41^, ^44^ Likewise, the omics study identified a defect in lung repair mechanisms,^123^ which the model did not address. Making these types of connections could be significant, particularly because tissue level changes are correlated with disease outcome.

## 6 MODELING THE POTENTIAL FOR UNIVERSAL VACCINES

Preventing influenza virus infections through vaccination is ideal. However, vaccines often lack efficacy because the virus mutates rapidly and novel viruses emerge through recombination. In addition, initiating a robust and long‐lasting response to the vaccine is challenging. Even when immunity is generated by a natural infection, long‐term protection may not be guaranteed.^138^ Furthermore, some evidence from mathematical and experimental studies suggests that viral epitopes may be masked from recognition by B cells,^29^, ^30^ which inhibits the generation of new antibodies during subsequent vaccinations or infections. The model and data were in agreement that the fold increase in antibody titer from baseline declines with repeated vaccination. This was due to an antigen dose threshold that depends on the level of pre‐existing antibodies and dictates the level of antibody boosting that can be attained. Sufficiently high antigen doses may be able to reduce the masking of antibodies.^30^ However, this could be difficult and may complicate protection by a universal vaccine, which aims to broadly protect against infection with any influenza virus subtype.

## 7 ANTIVIRAL THERAPY: THE CASE FOR IMMUNOMODULATORY DRUGS

Without effective vaccines, antivirals remain the primary measure for combatting influenza virus infections. The two major antivirals used to treat influenza are M2 inhibitors (M2Is) and NA inhibitors (NAIs).^66^ While M2Is disrupt ion‐channel activity of the M2 protein to limit virion uncoating inside the cell,^139^, ^140^ NAIs limit virus spread within the lung by preventing virions from being cleaved from infected cells and infecting new host cells.^139^ This reduces symptoms and slows disease progression, but does not significantly reduce the viral burden.^140^ Antiviral efficacy is greatest when the drug is administered prophylactically or within the first 24‐48 hours of symptom onset.^141^ Prophylaxis with NAIs has the most profound effect with a 2.5‐3.0 log_10_ reduction in viral loads.^66^, ^139^, ^142^ As discussed above, model analysis of viral kinetic models revealed that this is because the processes that the drugs target (ie, the viral life cycle) dominate only in the early stages of infection.^65^ Reduced efficacy and less than 1 log_10_ lower viral load are achieved if the drug is given in latter stages of infection (>3 days pi)^143^ when viral load kinetics are influenced predominantly by clearance mechanisms (eg, infected cell clearance (δ) and, to a lesser extent, virus clearance (c)).^65^

Estimates of antiviral efficacy can be obtained from simulating the model and altering the rate of virus production, $p(1-\varepsilon_{V})$, where $\varepsilon_{V}$ is the efficacy of the antiviral.^14^ Drug effectiveness is equal to 1 when the drug is 100% effective and 0 when the drug is inactive or absent. Model simulations suggest that targeting virus infection (β) would yield similar results as targeting virus production and that increased efficacy would be needed for an antiviral that improves clearance of free virus (*c*).^36^ Unsurprisingly, a therapy designed to improve the timing and/or rate of infected cell clearance (δ) could result in faster resolution.^36^

### 7.1 Detecting off target immune effects

A secondary effect of NAI therapy was detected in one study that assessed viral load kinetics when therapy was initiated either early or late in the infection.^36^ An extra term ($-\varepsilon_{T}T$) in the target cell equation together with the reduction in the rate of virus production ($p(1-\varepsilon_{v})$) was needed in the model to simultaneously capture the data^36^: $dT/dt=-\beta TV-\varepsilon_{T}T$, where $\varepsilon_{T}$ is the efficacy of the antiviral in reducing the number of cells susceptible to infection. The requirement of the $-\varepsilon_{T}T$ term in the model suggests that the antiviral limits the number of cells that can be infected. Indeed, this was independently observed in an experiment that assessed the area of the lung infected during therapy.^54^ Neither the model nor the viral load data identify the underlying mechanism. Interestingly, the predicted efficiency of this off‐target effect was significantly greater than the predicted efficacy of the antiviral inhibiting virus production (70% vs 10%).^36^ The lack of reduced viral loads even when fewer cells are infected 36 may suggest that infected cells are relatively long lived and that treatment does not shorten the time required to activate viral clearance mechanisms. The host variables that contribute to the reduced number of infected cells under NAI therapy remain unknown. Nevertheless, the reduced lung involvement would undoubtedly reduce symptoms, improve wound healing capabilities, and reduce subsequent comorbidities (eg, bacteria superinfection (discussed further below)).

### 7.2 Potential adverse consequences of antiviral therapy during virus coinfection

Although antivirals exist for treatment of influenza virus infection, antivirals targeting other coinfecting viruses (eg, RV, RSV, and PIV) have not been approved for use or are currently in development.^144^ Given that different virus pairings result in different outcomes (ie, infection enhancement or reduction), use of anti‐influenza therapy could result in beneficial or adverse consequences.^42^ In the case of IAV‐RSV coinfection, where influenza viruses reduce RSV growth,^104^, ^106^ antiviral therapy that limits IAV infection could inadvertently result in a resurgence of RSV replication. It's unclear if the off‐target effects of NAIs would be sufficient to facilitate or limit spread of the second virus. Conversely, in coinfections where disease is enhanced (eg, IAV‐RV coinfection^105^), anti‐influenza therapy could restrict the second virus from invading or significantly reduce coinfection‐related pathogenesis. This situation may have similarities to influenza‐bacterial coinfection, where antiviral therapy can lessen the synergism (discussed further below).^143^ Predicting outcomes from each of these scenarios are needed and ideal for investigation with mathematical models.

### 7.3 A role for antivirals and combination therapy in limiting bacterial coinfection

Because antivirals restrict viral growth and influenza disease severity, morbidity and mortality from invading bacterial pathogens can also be reduced.^143^ However, the time‐dependent efficacies observed during IAV infection are also reflected during bacterial coinfection, where NAI prophylaxis is more potent in reducing coinfection‐related mortality^143^ (A.M. Smith, unpublished data). The mechanisms underlying the improved outcome are currently unknown and may be a consequence of reduced pathogen burden and/or reduced inflammation. Antivirals can limit bacterial‐induced increases in virus production^126^ (A.M. Smith, unpublished data) and, thus, eliminate the post‐bacterial viral rebound (A.M. Smith, unpublished data). In addition, it is possible that NAI‐induced alterations to host responses have downstream consequences on the functionality of macrophages and neutrophils, which are critical for bacterial clearance. A diminished viral burden also minimizes the detrimental effect on aMΦs, which somewhat slows bacterial growth (A.M. Smith, unpublished data). Therapeutic manipulation of the aMΦ population has been examined experimentally 113 and mathematically (ie, $\hat{\phi }\left(V\right)\left(1-\varepsilon_{a}\right)$, where $\varepsilon_{a}$ is the efficacy of the therapy).^36^ As expected, bacterial burden and pneumonia were reduced.^113^ Although antibiotics have diminished efficacy during coinfection,^145^ analytical results suggest that combination therapy could increase the chances of successful immunotherapy or antiviral treatment by over 200%.^36^ This is because changes in the bacterial growth rate (*r*) also facilitates different outcomes of influenza‐bacterial coinfection.^36^ Similar to the degree of aMΦ depletion ($\hat{\phi }\left(V\right)$), the bacterial growth rate (*r*) is a bifurcation parameter and, thus, a drug target (eg, with protein synthesis inhibitors).^36^ However, the efficacy needed to sufficiently reduce bacteria through this class of drugs may be higher than immunomodulatory drugs.^36^

## 8 CONCLUDING REMARKS AND PERSPECTIVES

Influenza viruses continue to infect millions each year. Increased severity and case fatality rates due to secondary bacterial pneumonia have been emphasized by studies of the 1918, 1957, 1968, and 2009 influenza pandemics.^107^, ^109^, ^110^, ^146^ Influenza viruses that cause severe disease support higher incidence of bacterial coinfection, yet only a proportion of infections result in a coinfection.^94^, ^95^, ^96^ Furthermore, other respiratory viruses may also coinfect and enhance influenza‐related disease.^103^, ^105^, ^106^ Factors that impact influenza severity and, thus, coinfection risk are not well understood. Given that numerous viruses and bacteria can enhance influenza virulence and that two or more pathogens are often detected in individuals with pneumonia, understanding how different pathogens synergize is critical. Potentially even more important is discovering how antecedent viral or bacterial infections decrease influenza spread because the underlying mechanism(s) could be leveraged as drug therapy. However, knowledge about host immune control during influenza remains limited.

Mathematical models have been a key to evaluate host immune responses during influenza, disentangle factors that contribute to resolution and coinfection risk, and identify regulatory mechanisms ripe for drug targeting. In recent years, influenza models and techniques have improved together with better availability of data sets that measure numerous variables simultaneous and are sampled on frequent time scales. New imaging techniques have also facilitated a deeper understanding of the infection at the tissue level. This has allowed for robust development and parameterization of models, and verification of their accuracy in the laboratory. However, challenges still remain. The lack of non‐linear dynamics in some data (eg, constant viral loads during mid‐infection (Figure 2)) makes it challenging to assess specific host immune responses with a single data set. Incorporation of data from infections with modified experimental conditions (eg, low dose, aged hosts, coinfection, and/or antimicrobial therapy) should improve accuracy and reveal important dynamics that are not otherwise observable.

A substantial step in improving the quality of mathematical models lies with experimentally validating the models and their predictions, although few studies have taken this approach. Theoretical models yield a significant amount of insight some of which cannot be tested (eg, the rate of virus infection). This emphasizes the value of employing these methods in studying influenza virus infections and coinfections. However, designing experiments that test model‐derived hypotheses has proven critical to identifying host‐pathogen mechanisms and model accuracy. More studies of this nature should help refine the model formulations, allow for more in‐depth investigation of host responses, and limit misinterpretation of theoretical results. As technological advances continue to improve data quality and quantity and more data on viral–bacterial and viral–viral coinfections materializes, mathematical analyses like those described here will be critical.

## CONFLICT OF INTEREST

The author has no conflict of interest.
